# Disease activity in chronic inflammatory demyelinating polyneuropathy: association between circulating B-cell subsets, cytokine levels, and clinical outcomes

**DOI:** 10.1093/cei/uxad103

**Published:** 2023-08-28

**Authors:** Ayse Nur Ozdag Acarli, Erdem Tuzun, Elif Sanli, Gizem Koral, Ece Akbayir, Arman Cakar, Nermin Gorkem Sirin, Aysun Soysal, Fikret Aysal, Hacer Durmus, Yesim Parman, Vuslat Yilmaz

**Affiliations:** Neuromuscular Unit, Department of Neurology, Istanbul Faculty of Medicine, Istanbul University, Istanbul, Turkey; Department of Neuroscience, Aziz Sancar Institute of Experimental Medicine, Istanbul University, Istanbul, Turkey; Department of Neuroscience, Aziz Sancar Institute of Experimental Medicine, Istanbul University, Istanbul, Turkey; Department of Neuroscience, Aziz Sancar Institute of Experimental Medicine, Istanbul University, Istanbul, Turkey; Department of Neuroscience, Aziz Sancar Institute of Experimental Medicine, Istanbul University, Istanbul, Turkey; Neuromuscular Unit, Department of Neurology, Istanbul Faculty of Medicine, Istanbul University, Istanbul, Turkey; Neuromuscular Unit, Department of Neurology, Istanbul Faculty of Medicine, Istanbul University, Istanbul, Turkey; Department of Neurology, Bakirkoy Mazhar Osman Mental Health and Neurological Diseases Education and Research Hospital, Istanbul, Turkey; Department of Neurology, Bakirkoy Mazhar Osman Mental Health and Neurological Diseases Education and Research Hospital, Istanbul, Turkey; Department of Neurology, Bakirkoy Mazhar Osman Mental Health and Neurological Diseases Education and Research Hospital, Istanbul, Turkey; Neuromuscular Unit, Department of Neurology, Istanbul Faculty of Medicine, Istanbul University, Istanbul, Turkey; Neuromuscular Unit, Department of Neurology, Istanbul Faculty of Medicine, Istanbul University, Istanbul, Turkey; Department of Neuroscience, Aziz Sancar Institute of Experimental Medicine, Istanbul University, Istanbul, Turkey

**Keywords:** chronic inflammatory demyelinating polyneuropathy, B cell, cytokines, miRNA, skin biopsy, epidermal nerve fibers

## Abstract

Chronic inflammatory demyelinating polyneuropathy (CIDP), a common and treatable autoimmune neuropathy, is frequently misdiagnosed. The aim of this study is to evaluate the relationship between immunological markers and clinical outcome measures in a mixed cohort of patients with typical CIDP and CIDP variants at different disease stages. Twenty-three typical, 16 multifocal and five distal CIDP patients were included. Twenty-five sex and age-matched healthy controls and 12 patients with Charcot–Marie–Tooth type 1A (CMT1A) disease served as controls. Peripheral B-cell populations were analyzed by flow cytometry. *IL6, IL10, TNFA* mRNA and mir-21, mir-146a, and mir-155-5p expression levels were evaluated by real-time polymerase chain reaction in peripheral blood mononuclear cells (PBMC) and/or skin biopsy specimens. Results were then assessed for a possible association with clinical disability scores and intraepidermal nerve fiber densities (IENFD) in the distal leg. We detected a significant reduction in naive B cells (*P* ≤ 0.001), plasma cells (*P* ≤ 0.001) and regulatory B cells (*P* < 0.05), and an elevation in switched memory B cells (*P* ≤ 0.001) in CIDP compared to healthy controls. CMT1A and CIDP patients had comparable B-cell subset distribution. CIDP cases had significantly higher *TNFA* and *IL10* gene expression levels in PBMC compared to healthy controls (*P* < 0.05 and *P* ≤ 0.01, respectively). IENFDs in the distal leg showed a moderate negative correlation with switched memory B-cell ratios (*r* = –0.51, *P* < 0.05) and a moderate positive correlation with plasma cell ratios (*r* = 0.46, *P* < 0.05). INCAT sum scores showed a moderate positive correlation with *IL6* gene expression levels in PBMC (*r* = 0.54, *P* < 0.05). Altered B-cell homeostasis and *IL10* and *TNFA* gene expression levels imply chronic antigen exposure and overactivity in the humoral immune system, and seem to be a common pathological pathway in both typical CIDP and CIDP variants.

## Teaser

B-cell homeostasis is significantly altered in chronic inflammatory demyelinating polyneuropathy (CIDP) and is not reversed by immunomodulatory treatments. Charcot–Marie–Tooth type 1A has a comparable B-cell subset distribution with CIDP. Peripheral blood switched memory B-cell ratios correlate negatively with the severity of peripheral nerve damage in CIDP, whereas *IL6* gene expression levels show a positive correlation.

## Introduction

Chronic inflammatory demyelinating polyneuropathy (CIDP) is one of the most common and treatable chronic autoimmune neuropathies worldwide and is often misdiagnosed. A retrospective study reported that almost half of the patients incorrectly carry a diagnosis of CIDP [1]. The possible factors contributing to its misdiagnosis are the evaluation of ‘atypical’ CIDP and the placing of excessive diagnostic emphasis on subjective changes following immunotherapy. It is well known that the region and modality of involvement may vary among the variants of CIDP, as well as the treatment response and disease course (monophasic, relapsing, or progressive). Due to the complex pathophysiology of CIDP attributed to this clinical heterogenicity, CIDP still lacks potent molecules for diagnosis and disease monitoring [2]. Reliable biologic markers may help to reduce costs and side effects of unnecessary treatment, and also to prevent permanent functional disability due to secondary axonal degeneration.

The efficacy of intravenous immunoglobulin (IVIg) and plasma exchange in the treatment of CIDP suggests a pathogenetic contribution of humoral factors in the disease course, including autoantibodies. However, no pathogenic autoantibody has been detected so far in the vast majority of patients with CIDP. In a study, that aimed to determine the role of B cells in an animal model developing spontaneous autoimmune polyneuropathy (SAP), the authors developed a mutant B7-2^–/–^ mouse that was also null for mature B cells. B-cell deficiency in this strain prevented the development of SAP, which would indicate the pathogenic role of B-cells predominating over its regulatory role in this model [3]. B-cell activating factor (BAFF) overexpression in mice results in the survival of autoreactive B cells, thus leading to a breakdown of peripheral tolerance and the development of clinical autoimmune diseases [4]. BAFF, which is required for survival and maturation of B cells, was found to be increased in CIDP compared to healthy donors [4, 5]. Furthermore, IVIg treatment has been shown to decrease BAFF levels due to its anti-BAFF antibody content [5]. There is a very limited number of studies evaluating B-cell homeostasis in CIDP patients and collective data available so far are not sufficient to display a specific B-cell subset alteration in CIDP patients.

For this purpose, we performed peripheral B-cell phenotyping by flow cytometry, and assessed cytokine expression level alterations in peripheral blood and skin biopsy of CIDP patients by quantitative real-time polymerase chain reaction (qRT–PCR). We also investigated potential correlations between these immunological factors and clinical variables. Our findings imply the potential involvement of B cells in CIDP pathogenesis.

## Materials and methods

### Patients and controls

Patients diagnosed with CIDP at the Neuromuscular Unit of the Istanbul University, Istanbul Faculty of Medicine, Istanbul, Turkey, between 2016 and 2019 were prospectively enrolled in this study. Patients fulfilled diagnostic criteria according to the 2021 European Academy of Neurology/Peripheral Nerve Society (EAN/PNS) guidelines [6]. At entry, each patient underwent a neurological examination by the same physician. Patients were categorized as typical CIDP and CIDP variants. Typical CIDP was defined as progressive or relapsing, symmetric, proximal and distal muscle weakness of upper and lower limbs, and sensory involvement of at least two limbs with absent or reduced tendon reflexes in all limbs developing over at least 8 weeks. Patients, who had distal sensory loss and muscle weakness predominantly in the lower limbs were classified as distal CIDP. Patients with sensory loss and muscle weakness in a multifocal pattern (usually asymmetric, upper limb predominant, in more than one limb) were classified as multifocal CIDP [6]. The other CIDP variants (focal, motor, or sensory CIDP) were not included in the study as they were either infrequent in our CIDP patient cohort or did not fulfill the inclusion criteria of the study.

CIDP patients who had a concomitant medical history or clinical features suggestive of hereditary disease or other potential causes for polyneuropathy, including medications, toxins, and infections were excluded. Peripheral blood and skin biopsy samples were obtained on the day of IVIg treatment immediately before the infusion or on any day from patients taking daily oral glucocorticosteroids or immunosuppressive agents.

Age and sex-matched healthy controls, having no symptoms or risk factors for neuropathy and a normal neurologic examination, were recruited among hospital employers and their relatives. Since accompanying inflammatory incidents are established by experimental animal studies in Charcot–Marie–Tooth (CMT) disease [7], patients whose genetic testing was positive for CMT type 1A (CMT1A), were recruited as a disease control group. Subjects with a contraindication for skin biopsies such as anticoagulant treatment, known bleeding disorders or local skin infections were not included in the CIDP group. All study subjects’ sera were screened for antibodies against nodal and paranodal antigens such as contactin 1, contactin-associated protein receptor (CASPR) 1, neurofascin (NF)-155, and NF-186 (Unpublished results). The seropositive subjects were excluded from the study.

The study was approved by the Ethics Committee of the Istanbul University, Istanbul Medical Faculty (No. 2016-13-919 and 2018-18-1436) and each participant were enrolled after providing informed consent.

### Clinical evaluations

The clinical course of the disease was recorded and classified as monophasic, progressive, or relapsing–remitting. Monophasic illness fulfilled the clinical criteria for CIDP and then remained stable or improved for at least 6 months. Progressive disease worsened steadily showing no improvement with or without treatment up to the time of observation. The course was considered relapsing–remitting if at least two episodes of rapid clinical worsening were documented. The patient was classified as ‘untreated’ if at least 3 months passed from the last steroid or IVIg treatment and/or at least 6 months from the last immunosuppressive treatment. Functional disability was measured by Inflammatory Neuropathy Cause and Treatment (INCAT; range 0–10) [8] and Medical Research Council (MRC) sum score assessing six muscle pairs (range 0–60) [9, 10]. Disease activity was evaluated using the CIDP disease activity status (CDAS) [11]. Patients whose CIDP disease status was stable without treatment for less than 5 years according to CDAS were grouped as ‘remission’, and those who were stable with treatment for more than a year were considered to have ‘stable active disease’ or ‘improvement’. Patients with treatment for more than 3 months but less than a year were grouped as stable CIDP. Patients whose CIDP disease status was ‘unstable active disease’ were divided into two groups according to their treatment status. Patients with a progressive or relapsing–progressive course despite immune therapy of any duration were grouped as active CIDP with treatment. Patients who were treatment-naïve or untreated were grouped as active CIDP without treatment.

### Immunophenotyping by flow cytometry

Peripheral blood mononuclear cells (PBMCs) were isolated from blood samples with an EDTA tube by density gradient centrifugation using Ficoll–Paque, then resuspended in a freezing solution and stored in liquid nitrogen. Frozen PBMCs were thawed and washed, then stained with fluorescently labeled monoclonal antibodies [anti-human CD3-FITC, CD16/CD56-PE, CD45-PerCP, CD19-APC (Beckton Dickens (BD), MultitestTM), CD19-APC, CD27-FITC, CD24-PerCP, CD38-Alexa Fluor 700, IgD-APC/Cy7, and CD138-PE (Biolegend)] and, then six color immunofluorescence staining was utilized (BD FACS Aria II, Becton Dickinson, Franklin Lakes, NJ). Data were analyzed using Cell Quest (BD) and FlowJo7.6.5 software.

### Skin biopsy protocol

To determine cytokine expression levels and intraepidermal nerve fiber density (IENFD), two skin punch biopsies per subject were taken using a disposable 3-mm punch, under sterile conditions and topical anesthesia from the distal leg (10 cm above the lateral malleolus). Skin punch biopsies were taken unilaterally from the left side of all patients but from the affected side in multifocal CIDP patients. Skin biopsy specimens from the distal leg were used for ribonucleic acid (RNA) expression studies and processed for the IENFD as previously described [12] following quantification rules of European Federation of Neurological Societies/Peripheral Nerve Society (EFNS/PNS) guidelines [13, 14].

### Cytokine and microribonucleic acid (miRNA) expression (qRT–PCR) analysis

Total RNA was isolated from frozen PBMCs by using an isolation kit (RNeasy Mini Plus Kit, Catalog no: 74134, Qiagen, Hilden, Germany) and skin tissue samples were thawed on ice and homogenized in TRIzolTM Reagent (Cat no: 15596018, Invitrogen, Waltham, USA) using Bullet Blender Storm Pro homogenizer (Cat no: BBY24M, Next Advance, Troy, NY), then RNA was extracted by chloroform/phenol method with TRIzolTM. RNA quality was measured by the A260/A280 (1.9-2.1) and A260/A230 ratio (Thermo Scientific Nanodrop 2000^®^ Spectrophotometer).

Total RNA was then converted to complementary deoxyribonucleic acid (cDNA) by using the SCRIPT cDNA Synthesis Kit (Cat no: PCR-511S, Jena Bioscience, Jena, Germany) according to the manufacturer’s guideline. Q-PCR reactions were performed in Agilent Technologies MX3005P QPCR System by using SYBR green master mix (LightCycler^®^ 480SYBR Green I Master) and primers (shown in Supplementary Table S1) obtained from DNA Technology^®^. The relative messenger ribonucleotide acid (mRNA) expression levels were normalized to glyceraldehyde 3-phosphate dehydrogenase (GAPDH) expression.

The universal stem-loop qRT–PCR method was used for the quantification of mature miRNAs [15]. For the evaluation of miRNAs that affect the expression of these cytokines targeting B-cell [16–19] isolation of total miRNA was performed on frozen PBMC using the miRNeasy kit (Cat no: 217084, Qiagen, Hilden, Germany), following the manufacturer’s protocol. For the generation of miRNA-specific first-strand cDNA, total RNA was reverse transcribed using the Invitrogen SuperScriptTM II Reverse Transcriptase kit (Cat no: 18064014, Invitrogen, Waltham, USA) according to the manufacturer’s instructions by Bio-Rad CFX96 qPCR detection system in a reaction along with miR-specific stem-loop primers and U6 primers, as an internal reference gene. To determine expression levels of the corresponding miRNAs, cDNAs were PCR amplified by applying the miRNA and reference primer sets (shown in Supplementary Table S1) combined with LightCycler^®^ 480 SYBR Green I Master (Cat no: 04707516001, Roche, Basel, Switzerland) on Bio-Rad CFX96.

Relative quantifications of target gene levels were performed by the 2^–ΔΔCT^ method using the reference gene (2^−[ΔCT gene of interest − ΔCT GAPDH]^).

### Statistical analysis

According to the normality test results, analysis of multiple groups was performed by ANOVA followed by Tukey’s test as post hoc or Kruskal–Wallis tests followed by Dunn’s test as post hoc. Likewise, two group comparisons were performed with Student *t* or Mann–Whitney *U* tests. Nonparametric categorical data were compared with the chi-square test or Fisher’s exact test, as required. Correlation analyses were performed to identify possible linear association(s) between the B-cell immunophenotype, mRNA expression level, INCAT sum scores, MRC sum scores, and IENFDs in distal legs with mean values within the scope of the study. In this context, Pearson correlation metrics were determined between data categories. Strong or very strong correlations were defined as those with coefficients of 0.70–1.00, moderate correlations of 0.40–0.69, and weak as 0.10–0.39 [20]. All data in the figures are presented as mean ±  S.E.M., and *P*-values. Statistical analyzes and graphs were performed with SPSS IBM 21.0 and GraphPad Prism 5 programs, respectively. *P*-values < 0.05 were considered significant.

## Results

### Basic description of the cohort

Forty-four patients who fulfilled the 2021 EAN/PNS diagnostic criteria [6] for CIDP, 12 patients with CMT1A, and 25 healthy donors were enrolled in this study. All cases were negative for antibodies against nodal and paranodal antigens. CIDP group (remission, *n* = 11; stable, *n* = 21; active with treatment, *n* = 7; and active without treatment, *n* = 5) consisted of 23 (52%) typical CIDP, 16 (36%) multifocal CIDP, and 5 (12%) distal CIDP patients. Among 16 patients without treatment, 11 were in remission and five were in unstable active disease status. Only three CIDP patients were drug naïve. Among 28 CIDP patients with treatment, 21 patients were in stable CIDP while seven of them were in unstable active disease status. While 16 CIDP patients were grouped into untreated, six patients were with IVIg, 13 patients with steroids and nine patients with immunosuppressive treatment either with or without IVIg and/or steroids. Among nine CIDP patients with immunosuppressive treatment, six patients were taking azathioprine (three with steroids, one with IVIg, and one with both steroids and IVIg) while three patients were taking mycophenolate mofetil (two with steroids and one with IVIg). Sex and age distributions were similar between the CIDP, CMT1A, and control groups. Disease duration, mean age at disease onset, functional status assessed by MRC and INCAT, and treatment status were not different among typical CIDP and CIDP variants. The study participants’ demographic data and clinical features are detailed in Supplementary Table S2 and are summarized in Table 1.

**Table 1.** Demographic and clinical characteristics of study groups for distribution of peripheral blood mononuclear cell subsets

	CIDP (*N* = 44)	Typical CIDP (*N* = 23)	Multifocal CIDP (*N* = 16)	Distal CIDP (*N* = 5)	CMT1A (*N* = 12)	HC (*N* = 25)	*P* value
Sex (F/M)	17/27	9/14	5/11	3/2	7/5	12/13	NS
Age; years	40.8 ± 15.0	42.7 ± 16.9	36.9 ± 13.3	44.8 ± 10.0	35.3 ± 11.7	32.6 ± 5.8	NS
IENFD in the distal leg^a^	6.2 ± 3.4^a^	6.3 ± 3.4^a^	5.4 ± 3.0^a^	7.2 ± 5.2^a^	NA	10.4 ± 1.5^a^	0.0001^b^
Mean age of symptom onset; years	32.9 ± 15.3	34.2 ± 17.6	28.6 ± 11.8	40.2 ± 12.7	15.6 ± 10.2	NA	
Mean disease duration; months	113.6 ± 101.5	133.3 ± 116.3	103.3 ± 84.3	55.8 ± 32.8	237.0 ± 107.2	NA	
Treatment; n							
Steroid	13	8	4	1	NA	NA	
IVIg	6	2	3	1	NA	NA	
Immunosuppressive	9	4	3	2	NA	NA	
Untreated	16	9	6	1	NA	NA	
Disease Activity; *n*							
Remission	11	8	3		NA	NA	
Stable	21	12	5	4	NA	NA	
Active CIDP with treatment	5	2	3	0	NA	NA	
Active CIDP without treatment	7	1	5	1	NA	NA	

Note: Data expressed as mean±SD. CIDP; chronic inflammatory demyelinating polyneuropathy, CMT1A; Charcot–Marie–Tooth type 1A, F; female, HC; healthy control, IENFD; intraepidermal nerve fiber density, IVIg; intravenous immunoglobulin, M; male, NA; not applicable, NS; not significant.

^a^IENFD in the distal leg was analyzed in 19 CIDP patients (10 with typical CIDP, six with multifocal CIDP and three with distal CIDP) and in 10 healthy controls.

^b^CIDP vs HC.

### Reduced IENFD in CIDP patients compared to healthy controls

IENFDs in the distal leg were evaluated optimally in 19 CIDP patients and 10 healthy volunteers. Mean IENFD in the distal leg in CIDP (6.2 ± 3.4) was significantly reduced compared to healthy controls (10.4 ± 1.5; *P* = 0.0001). IENFD values are presented in Supplementary Table S2. Among those subjects, the skin biopsy findings and IENFD values of 16 CIDP patients and all 10 healthy volunteers were presented in detail in a previous report [12]. In summary, healthy subjects and clinically stable CIDP patients showed abundant epidermal nerves arising from subepidermal plexuses. However, in all unstable CIDP patients, epidermal nerve fibers were reduced or completely absent. Epidermal nerve fiber counts of typical CIDP and CIDP variants were similar to each other and both had significantly reduced IENFD compared to healthy controls (Table 1).

### Altered distribution of peripheral T-cell and B-cell subsets in CIDP

**Figure 1.**
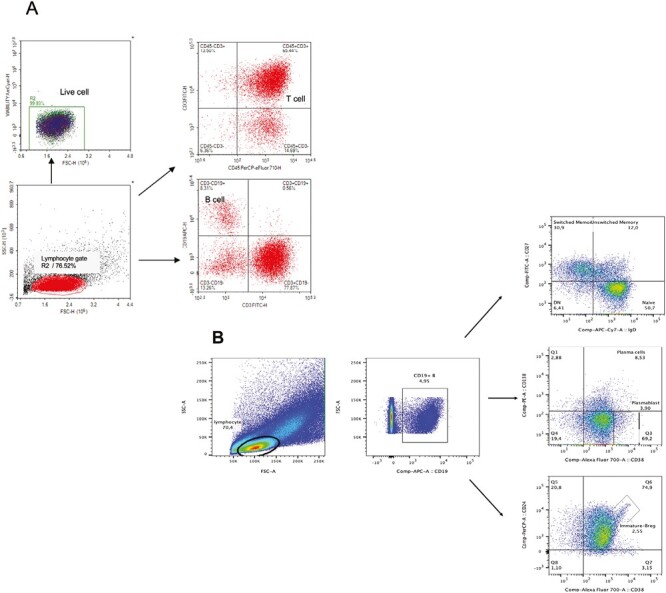
Analysis of T-cell and B-cell subtypes by flow cytometry. Cell subtypes in peripheral blood mononuclear cells (PBMCs) were identified using this gating strategy

The gating strategy of CD19 + B-cell subsets and T cells was shown in the representative figure. Total B cells in lymphocytes gate were divided into switched/unswitched memory, naïve, plasma, plasmablast, and immature/regulatory B (Breg) cells according to the surface expression of CD27, IgD, CD38, CD24, and CD138 (Fig. 1).

Peripheral immunophenotyping was performed in 44 CIDP patients, 12 CMT1A patients and 25 healthy volunteers (Table 1). Lymphocytes were gated on SSC/FSC dot-plot, then CD19 + B cell, CD3 + T cell, CD19 + CD38 + CD138 + plasma cells, CD19 + CD38++CD138 – plasmablast, CD19 + CD27 + memory, CD19 + IgD + CD27 – naïve, CD19 + IgD + CD27 + unswitched memory, CD19 + IgD-CD27 + switched memory, and CD19 + CD24++/+CD38++/+ Breg cells were evaluated.

**Figure 2.**
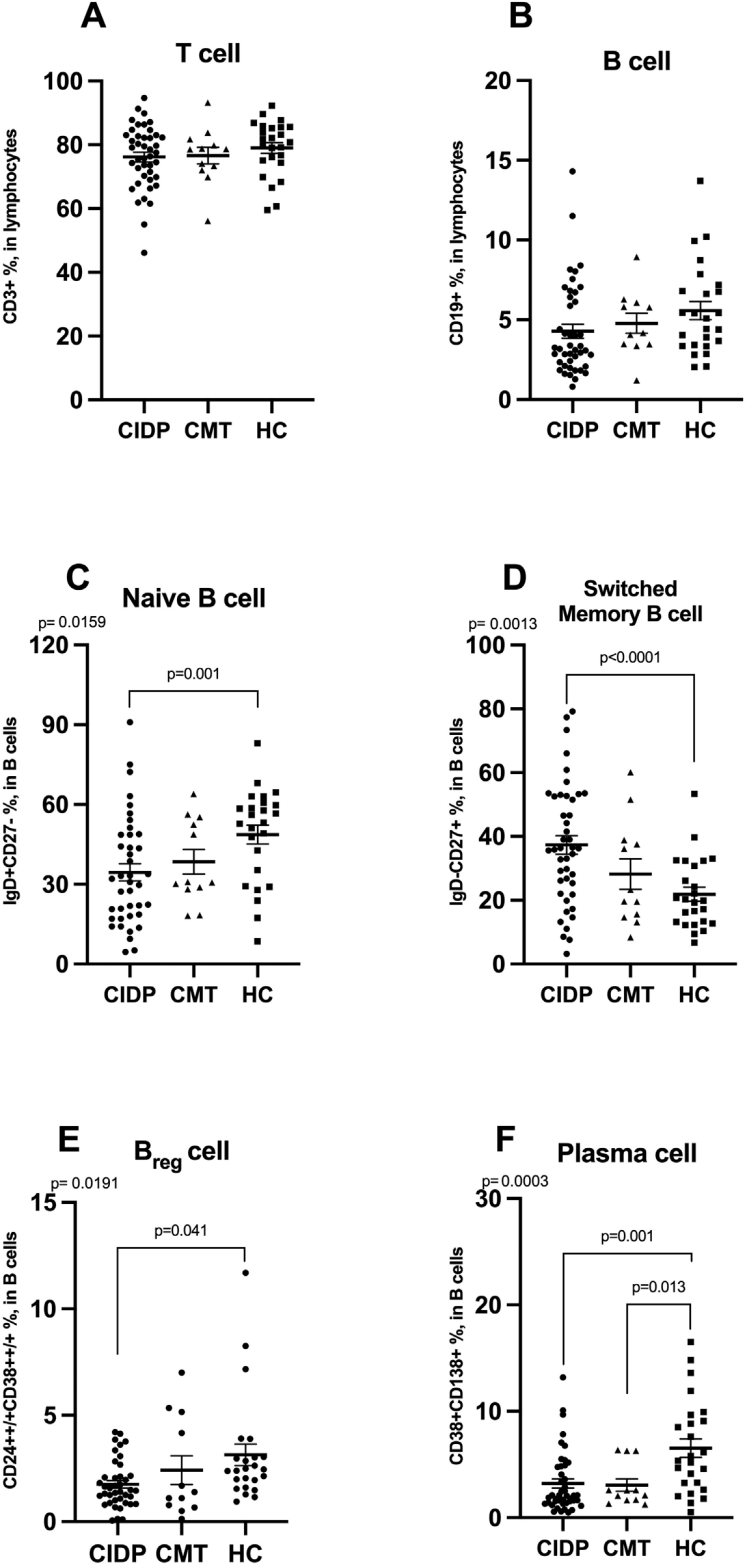
Distribution of frequencies (%) of total T cells, B cells and B-cell subtypes between patients with CIDP, CMT, and healthy controls. (A) Total T cells, (B) Total B cells, (C) Naïve B cells, (D) switched memory B cells, (E) Breg cells, and (F) plasma cells. Differences are calculated using the ANOVA, and values are provided as mean ±  S.E.M.. Breg cell; regulatory B cell, CIDP; chronic inflammatory demyelinating polyneuropathy, CMT; Charcot–Marie–Tooth, HC; healthy control

Compared to healthy controls, while T- and B-cell distribution was similar (Fig. 2A and 2B, respectively), CIDP patients had significantly reduced naive B cells (Fig. 2C) and had elevated switched memory B cells (Fig. 2D). Furthermore, Breg cells (Fig. 2E) and plasma cells (Fig. 2F) were also significantly decreased in the CIDP group compared to healthy controls. While there were no differences between CIDP and CMT patients in terms of B-cell subset ratios, CMT patients showed significantly decreased plasma cells (Fig. 2F) compared to healthy controls.

**Figure 3.**
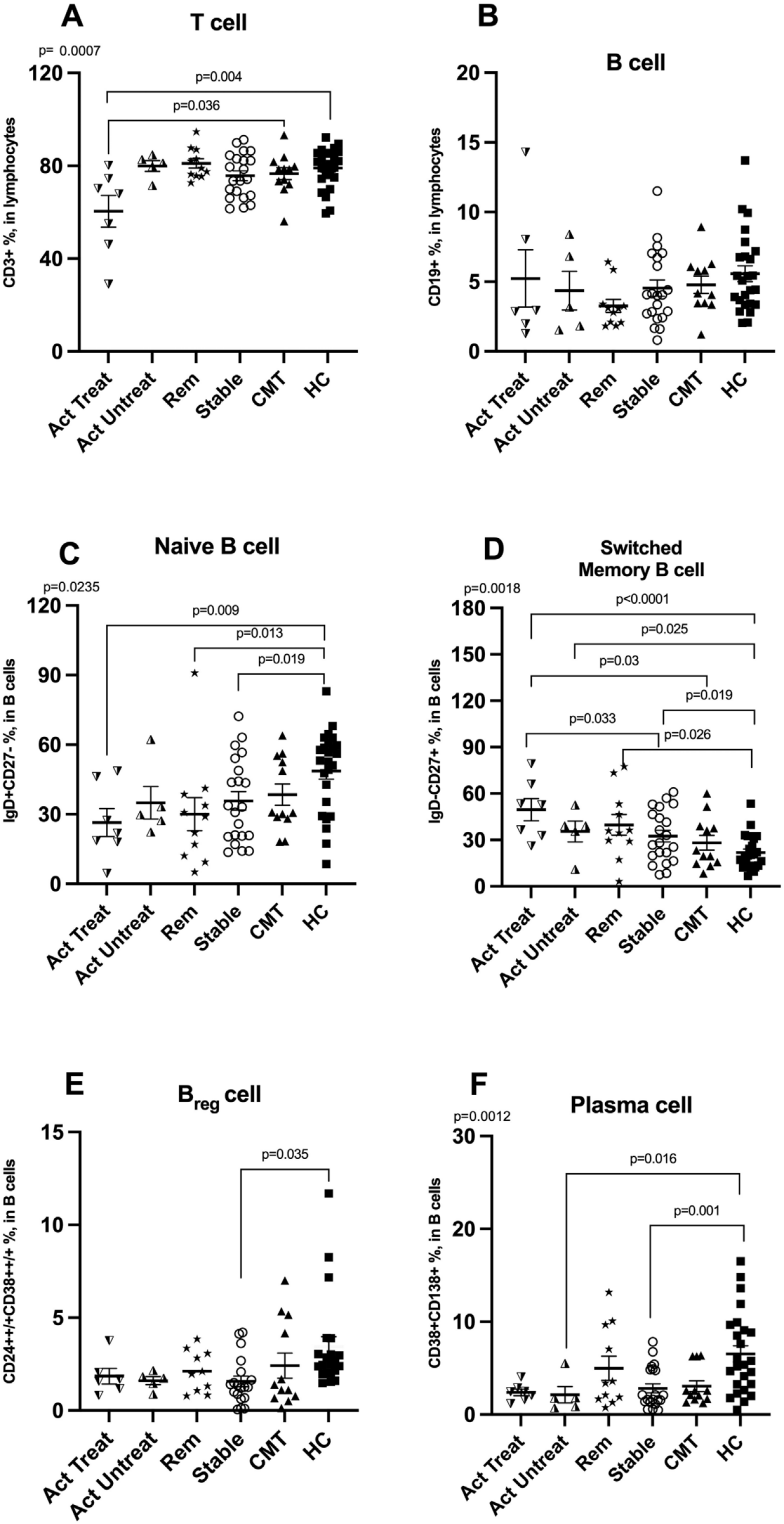
Distribution of frequencies (%) of total T cells, B cells and B-cell subtypes between CIDP subgroups according to disease activity status (Act Treat, Act Untreated, Rem, Stable), CMT and healthy controls. (A) Total T cells, (B) Total B cells, (C) Naïve B cells, (D) switched memory B cells, (E) Breg cells, and (F) plasma cells. Differences are calculated using the ANOVA, and values are provided as mean ±  S.E.M.. Breg cell; regulatory B cell, Act Treat; active CIDP with treatment, Act Untreat; active untreated CIDP, CIDP; chronic inflammatory demyelinating polyneuropathy, CMT; Charcot–Marie–Tooth, HC; healthy control, Rem; CIDP patients in remission, Stable; CIDP patients in stable disease status

No statistically significant differences were detectable in the frequencies of the B-cell subsets between typical CIDP and CIDP variants (data not shown). Since typical CIDP and CIDP variants have similar immunophenotypes, all CIDP patients were grouped according to CIDP disease status as described in the Materials and Methods. Active CIDP patients with treatment had significantly decreased frequencies of CD3 + T cells compared to CMT and healthy controls (Fig. 3A), while there was not any significant difference between the groups in terms of total B-cells‘ frequencies (Fig. 3B). Active CIDP with treatment, remission and stable CIDP groups had significantly decreased naïve B cells compared to healthy controls (Fig. 3C). Active CIDP patients with treatment had increased switched memory B cells compared to stable CIDP (Fig. 3D) and there was no significant difference in other B-cell subsets among CIDP subgroups according to disease status (Fig. 3E and F). Compared to CMT, active CIDP patients with treatment had increased switched memory B cells (Fig. 3D). Compared to healthy controls, all CIDP subgroups showed significantly increased switched memory B cells (Fig. 3D), and active CIDP without treatment and stable CIDP had reduced plasma cells (Fig. 3F). Although the percentages of Breg in each of the CIDP status forms were lower than in the healthy donors, the difference reached statistical significance in the stable group only (Fig. 3E). B-cell subsets among CIDP groups with different clinical courses (relapsing–remitting, monophasic, and progressive) were not found to be significantly different (data not shown).

**Figure 4.**
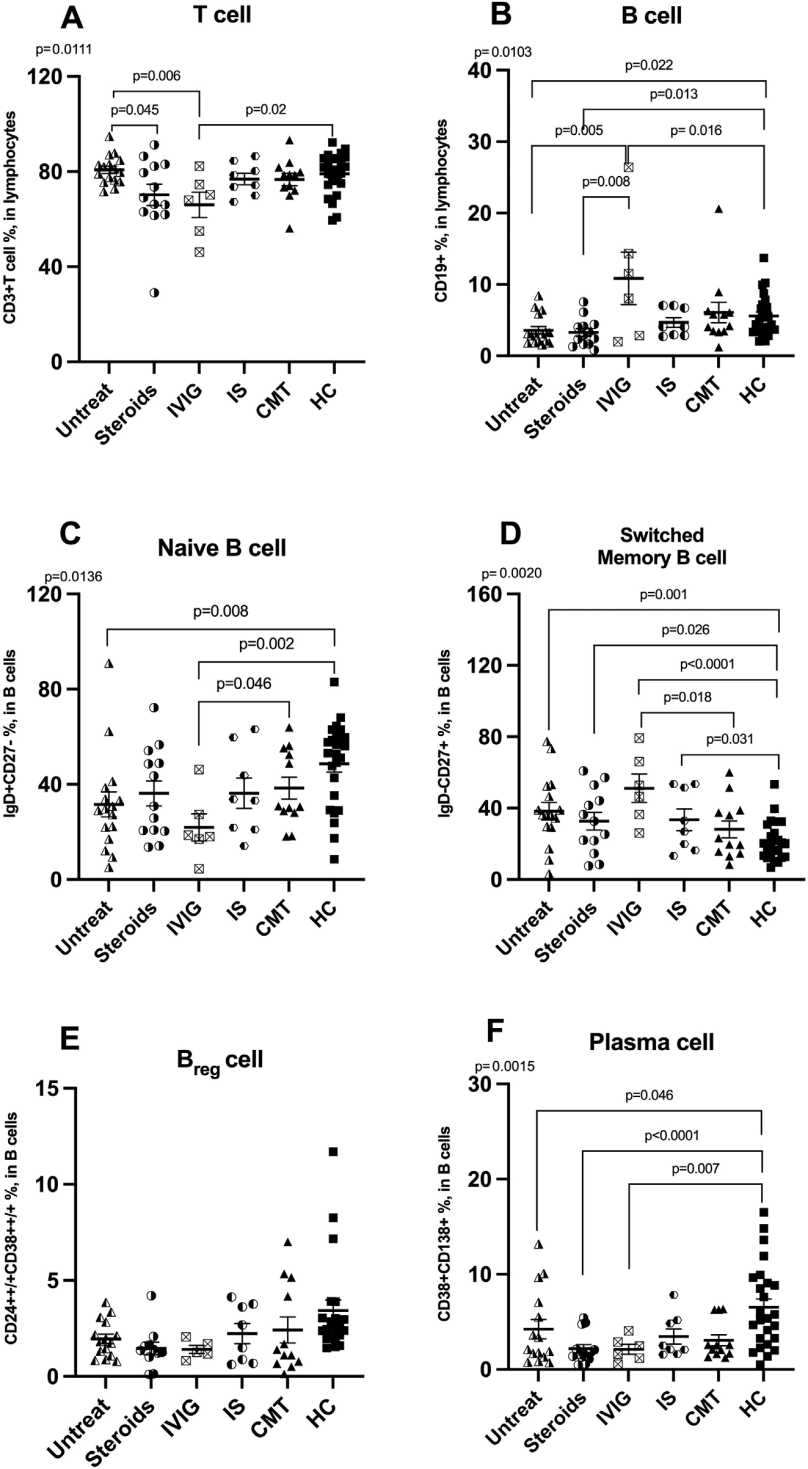
Distribution of frequencies (%) of total T cells, B cells and B-cell subtypes between CIDP subgroups according to treatment status (Untreated, Steroids, IVIG, and IS), CMT and healthy controls. (A) Total T cells, (B) Total B cells, (C) Naïve B cells, (D) switched memory B cells, (E) Breg cells, and (F) plasma cells. Differences are calculated using the ANOVA, and values are provided as mean ±  S.E.M.. Breg cell; regulatory B cell, CIDP; chronic inflammatory demyelinating polyneuropathy, CMT; Charcot–Marie–Tooth, HC; healthy control, IVIG; CIDP patients in intravenous immunoglobulin treatment, IS; CIDP patients in immunosuppressive treatment, Steroids; CIDP patients in steroid treatment, Untreat; untreated CIDP patients, HC; healthy control

When assessing whether immunomodulatory or immunosuppressive treatments could account for differences in lymphocyte count or lymphocyte subsets, there was a statistically significant decrease in the frequencies of CD3 + T cells in CIDP patients with IVIg treatment compared to untreated CIDP and healthy controls while CIDP patients with steroids had significantly decreased frequencies of CD3 + T cells compared to untreated CIDP (Fig. 4A). CIDP patients with IVIg treatment had significantly increased frequencies of CD19 + B cells compared to healthy controls and other CIDP patient treatment subgroups but CIDP patients with immunosuppressive treatment (Fig. 4B). However, CIDP patients without treatment and CIDP patients with steroid treatment had significantly decreased frequencies of CD19 + B cells compared to healthy controls (Fig. 4B). Patients with IVIg treatment had significantly reduced naïve B cells compared to CMT1A and healthy controls, while untreated CIDP patients had significantly decreased naïve B cells compared to healthy controls (Fig. 4C). Compared to healthy controls, all CIDP subgroups with any treatment and without treatment had significantly increased switched memory B cells while CIDP patients with IVIg treatment had significantly increased switched memory B cells compared to CMT (Fig. 4D). There was no significant difference in the frequencies of Breg cells between the groups (Fig. 4E). Untreated CIDP patients, CIDP patients with either steroids or IVIg groups had significantly reduced frequencies of plasma cells compared to healthy controls (Fig. 4F). In addition, Supplementary Table S3 shows percentages for all cell subtypes that we evaluated, even whose distribution did not differ between study groups. Values are presented in mean ± S.E.M.

### Increased interleukin 10 (*IL10*) and tumor necrosis factor-alpha (*TNFA*) gene expression levels in PBMC of CIDP patients compared to healthy controls

Cytokine gene expression analysis was performed in nine typical CIDP patients, nine CIDP variants (multifocal CIDP, *n* = 5; and distal CIDP, *n* = 4) and 10 healthy volunteers. The main characteristics of those subjects are provided in Table 2.

**Table 2.** Demographic and clinical characteristics of study groups for mRNA gene expression of cytokine in PBMC and skin biopsy

	CIDP (*N* = 18)	Typical CIDP (*N* = 9)	Multifocal CIDP (*N* = 5)	Distal CIDP (*N* = 4)	HC (*N* = 10)
Sex (F/M)	6/12	2/7	2/3	2/2	2/8
Age; years	40.1 ± 11.7	42.0 ± 10.7	35.0 ± 15.2	42.5 ± 9.9	31.0 ± 9.0
Mean age of symptom onset; years	34.9 ± 12.9	38.6 ± 13.3	28.6 ± 11.1	37.0 ± 12.1	NA
Mean disease duration; months	103.3 ± 70.5	108.1 ± 89.7	125.8 ± 41.3	64.3 ± 31.0	NA
Treatment; *n*					
Steroid	9	6	2	1	NA
Immunosuppressive	5	1	2	2	NA
Untreated	4	2	1	1	NA
Disease activity; *n*					
Remission	4	2	2	0	NA
Stable	12	7	2	3	NA
Active CIDP with treatment	1	0	1	0	NA
Active CIDP without treatment	1	0	0	1	NA

Note: Data expressed as mean±SD. CIDP; chronic inflammatory demyelinating polyneuropathy, F; female, HC; healthy control, M; male, NA; not applicable.

**Figure 5.**
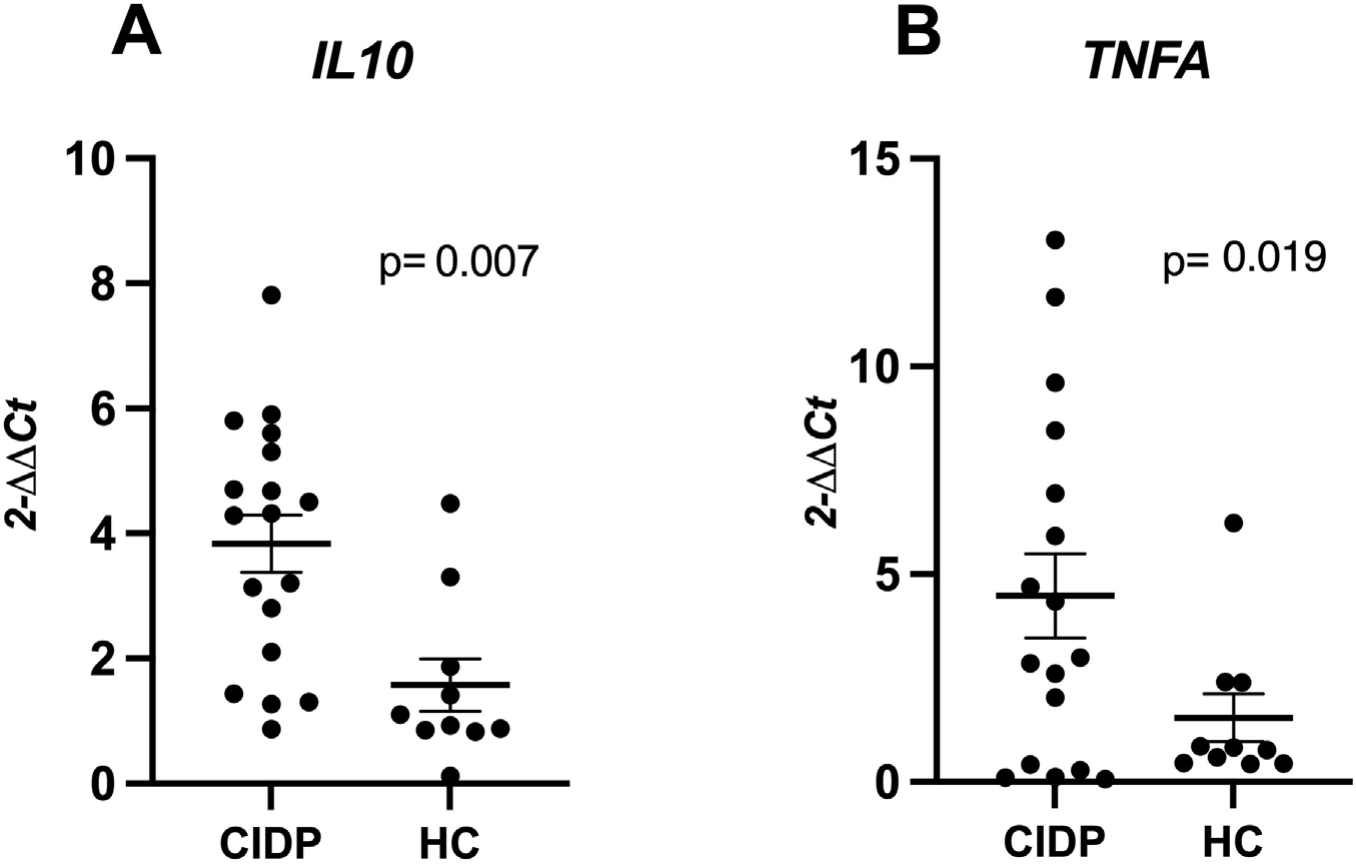
Gene expression levels of *IL10* and *TNFA* in CIDP and healthy controls. CIDP; chronic inflammatory demyelinating polyneuropathy, HC; healthy control

Firstly, *IL6, IL10*, and *TNFA* cytokine gene expression levels in PBMC and in skin biopsies were compared between typical CIDP, CIDP variants and healthy controls. Although there was no significant difference between CIDP patient subgroups (data not shown), *IL10 and TNFA* gene expression levels in PBMC were significantly increased in the combined CIDP cohort as compared to the healthy control group (Fig. 5A and B). There were no significant differences in *TNFA* gene expression levels in skin specimens. *IL10* gene expression was not detected in the skin biopsy specimens of study participants*. IL6* gene expression levels in PBMC and in skin biopsy were not different among CIDP and healthy controls. Likewise, there was no difference between CIDP groups stratified according to disease activity, treatment and course.

In addition, the results of all evaluated cytokine gene expression analyses are presented detail in Supplementary Table S4 with the cell percentages of all groups.

### miRNA expression levels show no difference between CIDP and healthy controls

hsa-miR-155-5p, hsa-miR-mir146a, and hsa-miR-21-5p expressions were deliberately selected for evaluation since they are known to most potently affect *IL6*, *IL10*, and *TNFA* expressions [21–23]. This analysis was performed in a limited number of patients and healthy controls listed in Table 2 with sufficiently available PBMC samples. There was no difference between study groups in any of these three miRNAs. However, typical CIDP patients tended to have increased mir-21 expression levels although this difference did not reach statistical significance (*P* = 0.0792). miRNA expression in PBMC and cytokine gene expression in skin samples were not different between the study groups, Therefore, we have presented all results in Supplementary Table S4.

### Correlation analyses

Possible correlation between the parameters evaluated within the study groups, IENFDs in the distal leg showed a moderate negative correlation with switched memory B cells (*r* = –0.51, *P* < 0.05) while showing a moderate positive correlation with plasma cells (*r* = 0.46, *P* < 0.05). INCAT sum scores showed a moderate positive correlation with *IL6* gene expression levels in PBMC (*r* = 0.54, *P* < 0.05). INCAT disability scores of the upper extremity also showed a moderate positive correlation with *IL6* gene expression levels in PBMC (*r* = 0.63, *P* ≤ 0.01), while showing a weak positive correlation with CD19 + B cells (*r* = 0.37, *P* ≤ 0.01). MRC sum scores showed a moderate negative correlation with *TNFA* gene expression levels in PBMC (*r* = –0.57, *P* < 0.05). Although the correlation *P* values did not stand Bonferroni correction, we have considered the correlations with high R coefficients worthy to mention. There was no correlation between age, disease duration, and lower extremity INCAT scores versus immunological variables.

Additionally, correlation visualization of all parameters is presented as a heatmap in the Supplementary Figure.

## Discussion

In the present study, we investigated changes in B-cell subsets and gene expression levels of B-cell-related cytokines in a mixed cohort of patients with typical CIDP and CIDP variants in different stages of disease activity compared to CMT1A and healthy control groups. We also investigated the effects of various immunomodulatory treatments on those biological markers in CIDP, as well as the correlations with clinical outcome measures. We found significantly increased switched memory B cells and decreased naïve, plasma cell, and Breg cell populations in the PBMC of CIDP patients as compared to healthy controls. These alterations were either not reversed or only mildly reversed by treatment and during remission. Overall, our results indicate a permanent disturbance in peripheral blood B-cell distribution in CIDP. Correlation studies imply that this disturbance might have an impact on the severity of the disease.

We found a significant reduction in plasma cells of CIDP patients with IVIg and steroid treatments, as well as untreated active CIDP patients, in contrast to two previous studies [24, 25]. The study of Mata and colleagues consisted of patients with various dysimmune neuropathies including CIDP patients [24], whereas the study of Kuhnke et al. reported markedly elevated plasma cell numbers in eight untreated CIDP patients and a significant decrease by IVIg treatment in six patients [25]. Similarly, our untreated CIDP patients showed relatively higher plasma cell ratios than steroid- and IVIg-treated patients. However, due to the high standard deviation of the untreated CIDP group and low statistical power, significance could not be attained. Thus, the difference in methodologies and a small number of study populations may have masked the plasma cell reduction in previous studies.

We detected a significant elevation in the percentage of switched memory B cells in CIDP compared to healthy controls similar to a previous report with acute inflammatory demyelinating polyneuropathy (AIDP) patients. In this previous study, memory B cells were positively correlated with clinical severity in AIDP patients. Furthermore, the percentage of memory B cells in patients with AIDP gradually decreased during the recovery phase [26]. In our study, memory B cells were not directly correlated with disability scores whereas they were negatively correlated with IENDs in distal leg. In our previous report, IENFDs in the distal leg were also negatively correlated with INCAT disability scores [12]. Different from AIDP patients, the percentages of memory B cells were still increased in CIDP patients with remission and stable disease status compared to healthy controls. While AIDP and CIDP may share common histopathological features, they are also known to display different disease courses. In contrast to AIDP which is a typically monophasic disorder, lacking clinical progression beyond 4 weeks, the persistence of increased memory B cells even in patients with remission, might be the contributing factor in the chronic course of the neuropathy [26]. This argument, which could serve to differentiate acute CIDP from AIDP and predict the disease course, needs to be further evaluated in larger cohorts. Interestingly, three previous studies reported no alteration in memory B cell frequencies in CIDP compared to healthy subjects [27–29]. However, two of them found that CIDP patients show lower Fcγ receptor IIB (FcγRIIB) expression levels on naive B cells and FcγRIIB expression levels fail to upregulate as B-cell progress from the naïve to the memory compartment compared to healthy controls [27, 28]. FcγRIIB is an inhibitory receptor preventing B cells with low affinity or self-reactive receptors from entering the germinal center and becoming IgG-positive plasma cells by transducing an inhibitory signal upon colligation with the B-cell receptor [30]. A potential underlying mechanism for the FcγRIIB dysregulation is a functionally relevant single nucleotide polymorphism in the FcγRIIB promoter, i.e., previously reported as being associated with systemic lupus erythematosus in humans and is significantly enriched in CIDP patients [27]. These data suggest that the dysregulation of this late B-cell differentiation checkpoint might lower the activation threshold for B cells, thereby leading to increased switching of naïve B cells to effector B cells. Since B cells do not infiltrate or locally accumulate in peripheral nerve lesions in CIDP [31, 32], the reduction of plasma cells in the peripheral blood might be a reflection of reduced migration of plasma cells from the germinal centers rather than a genuine decrease in absolute plasma cell counts in the immune system. Interestingly, a recent study showed elevated serum levels of CXC chemokine ligand 13 (CXCL13) in CIDP patients compared to healthy controls. CXCL13 is a B-cell recruiting factor i.e., constitutively expressed in follicular cells and macrophages in secondary lymphoid tissues and is essential for the development of secondary lymphoid organs. All of those findings may point to the B-cell activation in the secondary lymphoid organs in CIDP.

In our study, Breg cells are found to be significantly decreased in CIDP compared to healthy controls. In a mouse model of SAP that mimics CIDP, decreased levels of Bregs were found in the spleen and lymph nodes [3]. To our current knowledge, there are no previous studies evaluating Bregs in CIDP. Therefore, further studies are needed to confirm our findings and examine whether the reduction of Breg cells contributes to sustained peripheral nerve inflammation in CIDP.

The CD19 + B-cell percentages in untreated CIDP patients and CIDP patients with steroid treatment were significantly lower than in healthy controls, as previously reported [33]. However, the percentages of CD19 + B-cells in CIDP patients with IVIg treatment were significantly increased compared to untreated and healthy controls, as well as patients under steroid treatment, in contrast to the previous two studies including dysimmune neuropathy patients treated with IVIg [24, 25]. In one of them, the study cohort has also included patients with other inflammatory neuropathies other than CIDP. And in the other one, the CIDP patient group (*n* = 6) is tested within the first 3 days of the first IVIg treatment in contrast to our study, which evaluated PBMC on the last day of IVIg treatment interval, just before the next IVIg administration.

In our study, CD3 + T cells were significantly decreased in CIDP patients with IVIg or steroid treatment compared to the untreated group, as previously reported [24, 33]. By contrast, immune treatments failed to make a significant reversion in B-cell dysregulation in our CIDP patients. Furthermore, CIDP patients in the immunosuppressive treatment group showed no difference in the frequencies of B-cell subsets compared to other CIDP treatment subgroups. In almost all patients in the immunosuppressive treatment group, the immunosuppressive agent was in combination with steroid and/or IVIg. The heterogeneity and small number of this subgroup may have influenced the results. Notably, IVIg treatment appears to have caused an increase in effector memory B-cell ratios that were correlated with the degree of peripheral nerve damage. Given the significant involvement of B-cells in the development and continuity of autoimmunity, the use of B-cell targeting monoclonal antibodies in CIDP is more currently debated [34].

In contrast to a previous study [35], which reported decreased B-cell frequencies in CIDP patients with steroid treatment, there were no significant differences in B-cell frequencies between untreated and steroid-treated groups in our study. In the Klehmet study, all patients of the untreated group were clinically progressive in contrast to our study which included CIDP patients with both active clinical status and remission. Thus, the heterogeneity of disease activity among untreated patients may have changed the outcome in this direction.

Despite the significantly altered B-cell homeostasis, our patients were all negative for the known antibodies associated with CIDP. These findings prompted us to evaluate non-antibody-associated B-cell functions such as cytokine expression. The mRNA expression analysis revealed elevated *TNFA* and *IL10* gene expression levels in PBMC of CIDP patients while no significant difference was detected for *IL6*. Similar to our findings, previous studies showed unchanged gene expressions of IL-6 in blood or unaltered serum and CSF IL-6 levels in CIDP patients [36–38]. In our study, *IL6* gene expression levels showed a moderate positive correlation with disability scores and a weak positive correlation with the frequencies of CD19 + B-cells. Previous studies found an association between serum or gene expression levels of IL-6 and the duration or severity of polyneuropathy [39–41]. Thus, IL-6 might be reflecting nerve fiber degeneration in polyneuropathies, in accordance with our findings.

In our study, *TNFA* gene expression levels were increased and showed a moderate negative correlation with MRC disability scores indicating that CIDP patients with increased *TNFA* expression levels tended to display decreased MRC scores and thus increased disability. TNF-α exerts toxic effects on myelin and Schwann cells by increasing vascular permeability and breaking the blood-nerve barrier [42]. Compatible with our findings, some previous studies also reported increased serum TNF-α levels in CIDP by ELISA [33, 42, 43] and a relationship between disease severity and TNF-α levels [43].

IL-10 is known as an immunosuppressive cytokine and our findings support the results of previous studies that reported upregulated IL-10 expression in the peripheral nerves of patients with CIDP [44, 45] and elevated *IL10* production in PBMC in patients with active CIDP compared to healthy controls [46]. Intriguingly, in a mouse model, IL-10 overexpression has been shown to induce SAP. Furthermore, in another mouse model i.e., conducted with IL-10-deficient NOD.AireGW/+ mice, a significant delay has been exhibited in the onset of SAP [44]. Contrary to its anti-inflammatory effects, IL-10 activates B cells and promotes autoantibody secretion [47]. Sanvito et al. (2009) reported that P2, a myelin protein, elicited IL10 responses significantly more often in CIDP patients than in controls. All those data suggest an unexpected pathogenic role for IL-10 in autoimmune peripheral neuropathies. Further studies are needed to understand whether the increase of this cytokine is associated with increased switching of B cells, or a transitory epiphenomenon to reduce the ongoing inflammatory process.

Although IL-6 and TNF-α mRNA levels were previously found to be upregulated in nerve biopsies of CIDP patients [48, 49], we failed to show cytokine gene expression level alterations in skin biopsy samples of CIDP patients. This discrepancy might be due to a localized immunopathology in peripheral nerves or confounding effects of nonimmune skin cells such as keratinocytes and fibroblasts.

Expression levels of a panel of miRNAs regulating cytokine production were analyzed and no significant difference could be found among study groups. However, expression levels of miR-21 showed an increasing trend in CIDP patients. miR-21 is a well-characterized regulator of cytokines produced by B cells such as IL-10, IL-6, and TNF-α [21–23]. miR-21 has recently found to increase in white blood cells of patients with polyneuropathies when compared to healthy controls [50]. miR-21 is also a known inducer of IL-10 expression [51] i.e., found to be elevated in our CIDP patient cohort. Thus, our results lend to support the involvement of miR-21 and associated cytokines in CIDP pathogenesis.

We also compared B-cell homeostasis and cytokine gene expression levels between typical CIDP and CIDP variants aiming to investigate a possible difference in the pathogenesis of these subtypes. Even two recent studies reported immunologic differences in the repertoire and function of T cells in typical CIDP versus atypical CIDP [52, 53], we did not detect any significant difference between CIDP subtypes in terms of B-cell immunophenotypes and cytokine gene expression levels, as reported in a previous study [29]. This controversy may be derived from the differences in patients populations in atypical CIDP variant groups in those studies compared to our study, as we have checked and have excluded patients with nodal/paranodal antibodies. Furthermore, the study group in Staudt et al. consisted of only CIDP patients with active disease status [52] in contrast to our mixed patient group. Nevertheless, our finding may also reflect a common B-cell-related pathway involvement in CIDP and variants.

Another notable finding was that patients with CMT, an untreatable hereditary polyneuropathy, showed similar B-cell immunophenotype alterations as CIDP patients. Peripheral nerve of CMT1A patients is well known to exhibit inflammatory cells and inflammation is known to play a role in the physiopathology of CMT [54, 55] Also, clinical features of CMT may occasionally mimic those of CIDP [56]. Overall, these results suggest that B-cell alterations observed in our CIDP cohort are not unique to CIDP and putatively occur in response to the ongoing inflammation and autoimmunity of the peripheral nerves. This assertion needs to be further supported by future studies.

The major limitation of our study is the lack of follow-up, therefore it can not answer whether the alterations in B cells and cytokine expressions are the cause or consequence of CIDP. Furthermore, the heterogeneity of patient characteristics such as disease subtypes, disease, and treatment durations and treatment modalities resulted in a high number of study subgroups with a low number of patients. Another limitation was the absence of skin biopsies from a disease control group for the comparison of cytokine gene expressions.

Despite these limitations, we have shown for the first time an alteration of circulating B-lymphocyte subsets in CIDP patients, including a naïve, plasma and regulatory B-cell reduction and increased proportions of switched memory B cells, and there was no tendency to normalization after treatment with IVIg, steroids or immunosuppressive agents. Moreover, peripheral blood memory B-cell ratios and *IL6* expression levels were found to be associated with the severity of peripheral nerve damage. B-cell-mediated immune responses might play a crucial role in the etiopathogenesis of CIDP and represent important pharmacological targets in patients with CIDP.

## Supplementary Material

uxad103_suppl_Supplementary_Figure

uxad103_suppl_Supplementary_Tables

## Data Availability

The data underlying this article will be shared on reasonable request to the corresponding author.

## Abbreviations:

AIDP: acute inflammatory demyelinating polyneuropathy
BAFF: B-cell activating factor
Breg: regulatory B cell
CASPR: contactin associated protein
CDAS: CIDP disease activity status
cDNA: complementary deoxyribonucleic acid
CIDP: chronic inflammatory demyelinating polyneuropathy
CMT1A: Charcot–Marie–Tooth type 1A
CXCL3: CXC chemokine ligand 13
EAN/PNS: European Academy of Neurology/Peripheral Nerve Society
EFNS/PNS: European Federation of Neurological Societies/Peripheral Nerve Society
FcγRIIB: Fcγ receptor IIB
GAPDH: glyceraldehyde 3-phosphate dehydrogenase
IENFD: intraepidermal nerve fiber density
IL: interleukin
INCAT: Inflammatory Neuropathy Cause and Treatment
IVIg: intravenous immunoglobulin
miRNA: micro-ribonucleic acid
MRC: Medical Research Council
mRNA: messenger ribonucleic acid
NF: neurofascin
PBMC: peripheral blood mononuclear cell
qRT–PRC: quantitative real time polymerase chain reaction
RNA: ribonucleic acid
S.E.M.: standard error of mean
SAP: spontaneous autoimmune polyneuropathy
TNF: tumor necrosis factor.

## Permission to Reproduce

The authors report that there is no reproduced material from other sources in this article.

## Clinical Trial Registration

The authors report that this study is not a clinical trial.
