# Supplementary figures and images for: Disease activity in chronic inflammatory demyelinating polyneuropathy: association between circulating B-cell subsets, cytokine levels, and clinical outcomes
Source: Clin Exp Immunol. 2023 Aug 28;215(1):65–78. doi: 10.1093/cei/uxad103 (PMC10776240; doi:10.1093/cei/uxad103)

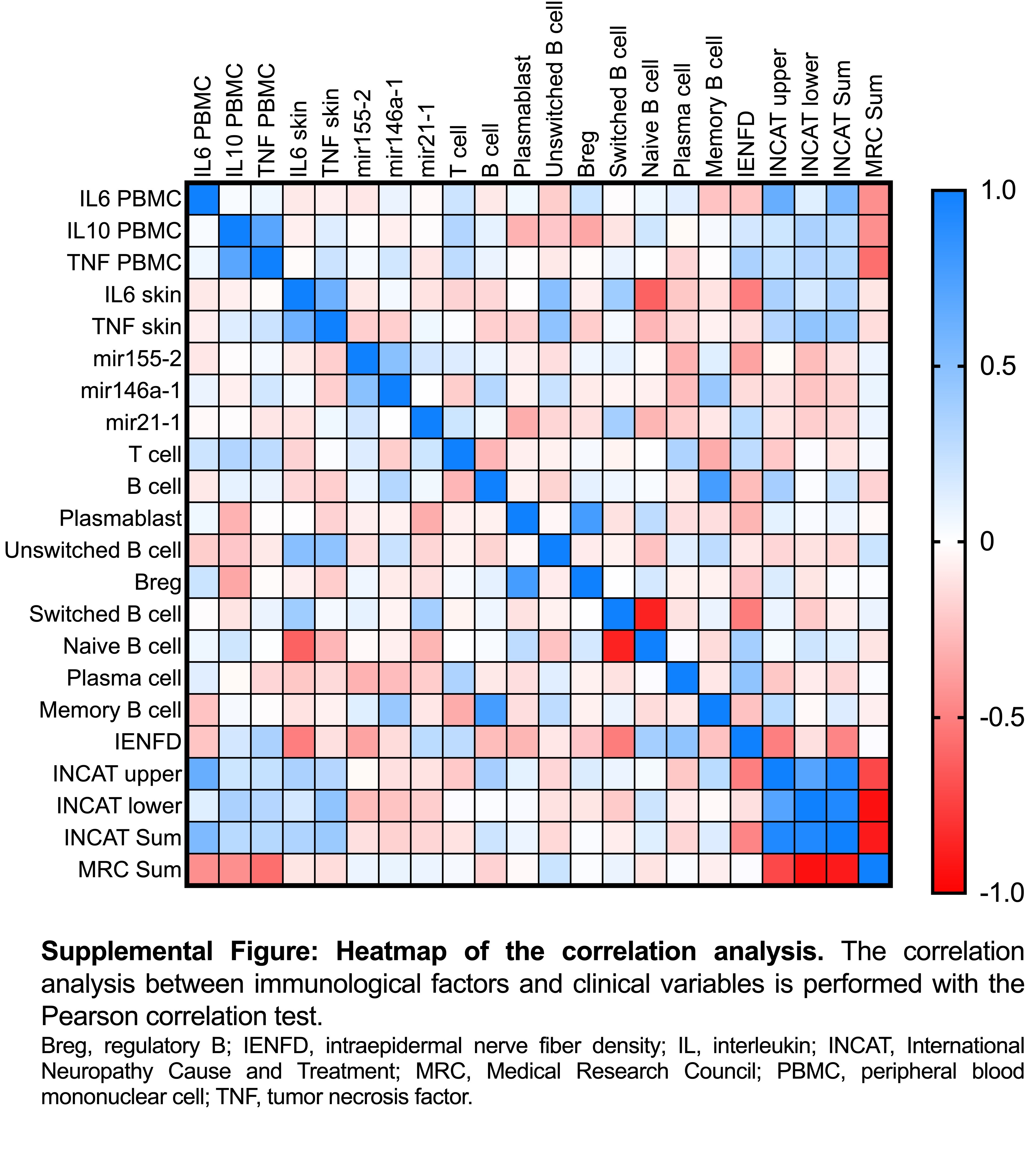

Supplement: uxad103_suppl_Supplementary_Figure [file uxad103_suppl_supplementary_figure.jpeg]
