# Supplementary material for: Disease activity in chronic inflammatory demyelinating polyneuropathy: association between circulating B-cell subsets, cytokine levels, and clinical outcomes
Source: Clin Exp Immunol. 2023 Aug 28;215(1):65–78. doi: 10.1093/cei/uxad103 (PMC10776240; doi:10.1093/cei/uxad103)
Supplement: uxad103_suppl_Supplementary_Tables [file uxad103_suppl_supplementary_tables.docx]

**Supplemental Table 1**. Target primers and their sequences used in reverse transcription reaction and qPCR. The same universal reverse primer was used for 3 different miRNAs

| **Targets** | **Primer type** | **Primer sequences (5'→3')** |
| --- | --- | --- |
| IL6 | *Forw primer* | ATTCCGGGAACGAAAGAGAA |
|  | *Rev primer* | TCTTCTCCTGGGGGTACTGG |
| IL10 | *Forw primer* | CATCGATTTCTTCCCTGTGAA |
|  | *Rev primer* | TCTTGGAGCTTATTAAAGGCATTC |
| TNFα | *Forw primer* | CCCAGGGACCTCTCTCTAATC |
|  | *Rev primer* | ATGGGCTACAGGCTTGTCACT |
| GAPDH | *Forw primer* | CCATCAATGACCCCTTCATT |
|  | *Rev primer* | TTGACGGTGCCATGGAATTT |
| hsa-miR-155-5p | *Stem-loop RT primer* | GTTGGCTCTGGTGCAGGGTCCGAGGTATTCGCACCAGAGCCAACAACCCC |
|  | *Forw primer* | CCGGCTTAATGCTAATCGTGATA |
| hsa-miR-mir146a | *Stem-loop RT primer* | GTTGGCTCTGGTGCAGGGTCCGAGGTATTCGCACCAGAGCCAACAACCCA |
|  | *Forw primer* | GGCCGTGAGAACTGAATTCC |
| hsa-miR-21-5p | *Stem-loop RT primer* | GTTGGCTCTGGTGCAGGGTCCGAGGTATTCGCACCAGAGCCAACTCAACA |
|  | *Forw primer* | CGGGCTAGCTTATCAGACTG |
| Universal | *Rev primer* | GTGCAGGGTCCGAGGTATT |
| U6 | *Stem-loop RT primer* | CGCTTCACGAATTTGCGTGTCA |
|  | *Forw primer* | GCTTCGGCAGCACATATACTAAAAT |
|  | *Rev primer* | CGCTTCACGAATTTGCGTGTCAT |

**Supplemental Table 2**. Demographic data, clinical features and intraepidermal nerve fiber densities in patients with chronic iflammatory demyelinating polyneuropathy

| **Patient No** | **Sex** | **Age** | **Diagnosis** | **Course** | **Treatment** | **CDAS** | **INCAT** | | | **MRC**  **sum score** | **IENFD in distal leg (fibers/mm)** |
| --- | --- | --- | --- | --- | --- | --- | --- | --- | --- | --- | --- |
|  |  |  |  |  |  |  | **Upper extremity** | **Lower Extremity** | **Total score** |  |  |
| 1 | F | 54 | Distal CIDP | R | Steroid | 3B | 1 | 0 | 1 | 60 | 1,2 |
| 2 | M | 30 | Distal CIDP | Mo | Steroid + MM | 3B | 0 | 1 | 1 | 54 | 10,9 |
| 3 | F | 44 | Distal CIDP | Mo | AZA | 3B | 0 | 0 | 0 | 60 | 9,4 |
| 4 | F | 54 | Distal CIDP | Mo | IVIg | 3B | 0 | 0 | 0 | 60 |  |
| 5 | M | 42 | Distal CIDP | P | Untreated | 5B | 0 | 0 | 0 | 60 |  |
| 6 | M | 16 | Multifocal CIDP | R | Steroid | 3A | 0 | 0 | 0 | 60 | 7,9 |
| 7 | F | 37 | Multifocal CIDP | R | Steroid | 5C | 1 | 0 | 1 | 60 | 4,1 |
| 8 | M | 27 | Multifocal CIDP | R | Steroid + AZA | 4B | 1 | 1 | 2 | 60 | 3,5 |
| 9 | F | 57 | Multifocal CIDP | P | IVIg + MM | 5C | 4 | 1 | 5 | 51 | 4,9 |
| 10 | M | 38 | Multifocal CIDP | R | Untreated | 2A | 0 | 0 | 0 | 60 | 10,1 |
| 11 | F | 39 | Multifocal CIDP | R | Untreated | 5C | 0 | 0 | 0 | 60 | 2,0 |
| 12 | M | 42 | Multifocal CIDP | P | IVIg | 5C | 4 | 0 | 4 | 52 | 1,7 |
| 13 | M | 25 | Multifocal CIDP | R | Steroid + IVIg + AZA | 3B | 0 | 1 | 1 | 46 | 7,3 |
| 14 | F | 29 | Multifocal CIDP | Mo | Untreated | 2A | 0 | 0 | 0 | 60 |  |
| 15 | M | 41 | Multifocal CIDP | Mo | Steroid | 4B | 0 | 0 | 0 | 60 |  |
| 16 | F | 40 | Multifocal CIDP | R | Untreated | 5A | 3 | 2 | 5 | 48 |  |
| 17 | M | 34 | Multifocal CIDP | R | Untreated | 5A | 1 | 0 | 1 | 60 |  |
| 18 | M | 62 | Multifocal CIDP | P | IVIg | 5C | 1 | 1 | 2 | 59 |  |
| 19 | M | 22 | Multifocal CIDP | Mo | Untreated | 2B | 0 | 0 | 0 | 60 |  |
| 20 | M | 24 | Multifocal CIDP | R | IVIg | 4B | 2 | 1 | 3 | 52 |  |
| 21 | M | 57 | Multifocal CIDP | R | Steroid | 3B | 1 | 1 | 2 | 59 |  |
| 22 | F | 70 | Typical CIDP | Mo | Untreated | 2A | 0 | 0 | 0 | 60 | 8,2 |
| 23 | M | 26 | Typical CIDP | R | Steroid | 3A | 0 | 0 | 0 | 60 | 4,4 |
| 24 | M | 33 | Typical CIDP | R | Steroid | 3B | 1 | 1 | 2 | 60 | 3,6 |
| 25 | M | 39 | Typical CIDP | Mo | Steroid | 3B | 1 | 1 | 2 | 60 | 3,3 |
| 26 | M | 41 | Typical CIDP | Mo | Steroid | 3A | 0 | 0 | 0 | 60 | 11,4 |
| 27 | M | 74 | Typical CIDP | Mo | Steroid + AZA | 3B | 1 | 0 | 1 | 60 | 8,4 |
| 28 | M | 41 | Typical CIDP | R | Untreated | 2B | 0 | 0 | 0 | 60 | 10,8 |
| 29 | F | 48 | Typical CIDP | Mo | Steroid | 3B | 1 | 0 | 1 | 60 | 4,0 |
| 30 | M | 33 | Typical CIDP | R | Untreated | 2B | 0 | 0 | 0 | 60 |  |
| 31 | F | 46 | Typical CIDP | Mo | Steroid | 3B | 1 | 0 | 1 | 60 |  |
| 32 | M | 62 | Typical CIDP | Mo | Untreated | 2A | 0 | 0 | 0 | 60 |  |
| 33 | F | 62 | Typical CIDP | R | Steroid + MM | 3B | 3 | 2 | 5 | 54 |  |
| 34 | M | 41 | Typical CIDP | R | Untreated | 2B | 0 | 0 | 0 | 60 |  |
| 35 | F | 68 | Typical CIDP | Mo | Untreated | 2A | 0 | 0 | 0 | 60 |  |
| 36 | F | 18 | Typical CIDP | P | IVIg | 5C | 4 | 5 | 9 | 24 |  |
| 37 | M | 17 | Typical CIDP | P | Untreated | 5B | 4 | 5 | 9 | 30 |  |
| 38 | F | 43 | Typical CIDP | P | IVIg | 5C | 3 | 5 | 8 | 24 |  |
| 39 | M | 12 | Typical CIDP | Mo | Steroid | 3B | 0 | 0 | 0 | 60 |  |
| 40 | F | 37 | Typical CIDP | R | IVIg + AZA | 4B | 3 | 1 | 4 | 52 |  |
| 41 | M | 41 | Typical CIDP | R | Steroid + AZA | 4B | 0 | 1 | 1 | 60 |  |
| 42 | M | 48 | Typical CIDP | R | Steroid | 3A | 0 | 0 | 0 | 60 |  |
| 43 | F | 71 | Typical CIDP | Mo | Untreated | 2B | 0 | 0 | 0 | 60 |  |
| 44 | M | 39 | Typical CIDP | Mo | Untreated | 2A | 0 | 0 | 0 | 60 |  |

AZA, azathioprine; CDAS, CIDP disease activity status; CIDP, chronic inflammatory demyelinating polyneuropathy; F, female; IENFD, intraepidermal nerve fiber density; INCAT, International Neuropathy Cause and Treatment; IVIg, intravenous immunoglobulin; M, male; MM, mycophenolate mofetil; Mo, Monophasic; MRC, Medical Research Council; P, progressive; R, relapsing-remitting.

**Supplemental Table 3.** Cell percentages of all groups

|  |  | **T-cell** | **B-cell** | **Naive B-cell** | **Switched**  **B-cell** | **Breg cell** | **Plasma cell** | **Plasmablast** | **Memory** | **Unswitched B-cell** |
| --- | --- | --- | --- | --- | --- | --- | --- | --- | --- | --- |
| **Mean ± S.E.M.** | N | **CD3+ % in lymp** | **CD19+ % in lymp** | **IgD+CD27- % in B-cells** | **IgD-CD27+ % in B cells** | **CD24++/+CD38++/+ % in B-cells** | **CD38+CD138+ % in B-cells** | **CD38++CD138- % in B-cells** | **CD19+CD27+ % in lymp** | **IgD+CD27+ % in B-cells** |
| **CIDP** | 44 | 76.2±1.52 | 4.29±0.440 | 34.49±3.21 | 37.34±2.843 | 1.756±0.175 ***** | 3.22±0.431 ***** | 3.47±0.315 | 2.69±0.278 | 18.2±1.46 |
| ***Active with treatment*** | 5 | 60.4±6.85 | 5.23±2.06 | 26.5±6.01 | 49.5±7.20 | 1.85±0.422 | 2.39±0.353 | 3.68±0.766 | 3.92±1.22 | 12.6±2.01 |
| ***Active without treatment*** | 7 | 80.0±2.27 | 4.35±1.38 | 35.0±7.04 | 35.4±6.76 | 1.61±0.215 | 2.14±0.873 | 2.89±0.401 | 2.32±0.619 | 17.4±3.85 |
| ***Remission*** | 11 | 75.7±2.08 | 3.25±0.467 | 35.8±3.96 | 32.5±3.68 | 1.57±0.289 | 2.82±0.482 | 3.33±0.493 | 2.78±0.336 | 20.6±2.3 |
| ***Stable*** | 21 | 81.0±2.00 | 4.54±0.581 | 30.0±7.08 | 39.7±6.66 | 2.12±0.352 | 4.98±1.3 | 3.85±0.702 | 1.91±0.316 | 17.5±2.98 |
|  |  |  |  |  |  |  |  |  |  |  |
| ***Untreated*** | 16 | 80.7±1.51 | 3.6±0.526 | 31.6±5.24 | 38.4±4.94 | 1.95±0.248 | 4.25±1.03 | 3.55±0.503 | 2.03±0.283 | 17.4±2.3 |
| ***Steroids*** | 13 | 70.2±4.42 | 3.28±0.544 | 36.2±5.23 | 32.8±4.97 | 1.45±0.328 | 2.19±0.432 | 3.57±0.598 | 2.26±0.336 | 21.4±3.06 |
| ***IVIg*** | 6 | 66±5.4 | 10.8±3.67 | 22±5.7 | 51.2±7.9 | 1.4±0.215 | 2.12±0.519 | 3.51±0.931 | 4.51±1.3 | 10.9±2.12 |
| ***Immunosuppressive*** | 9 | 76.9±2.41 | 4.68±0.688 | 36.3±6.37 | 33.5±6.06 | 2.23±0.526 | 3.46±0.798 | 3.18±0.892 | 3.1±0.661 | 20.6±3.22 |
| **CMT** | 12 | 76.7±2.58 | 4.78±0.621 | 38.47±4.567 | 28.18±4.738 | 2.422±0.673 | 3.06±0.582 | 3.07±0.566 | 2.63±0.335 | 18.2±4.41 |
| **HC** | 25 | 79.0±1.72 | 5.58±0.569 | 48.68±3.544 | 21.89±2.199 | 3.138±0.511 | 6.54±0.878 | 4.81±0.708 | 3.15±0.353 | 20.2±1.96 |

Breg, regulatory B; CIDP, chronic inflammatory demyelinating polyneuropathy; CMT, Charcot-Marie-Tooth disease; HC, healthy control; IVIg, intravenous immunoglobulin; lymp, lymphocyte; S.EM., standard error of mean.

**Supplemental Table 4.** Gene expression levels of *IL6, IL10* and *TNFA,* and miRNA expression levels of all groups

| **2-ddCt, Mean ± SD** | **Typical CIDP (N=9)** | **Multifocal CIDP (N=5)** | **Distal CIDP (N=4)** | **HC**  **(N=10)** |
| --- | --- | --- | --- | --- |
| ***IL6*, in PBMC** | 3.34±2.58 | 3.95±3.32 | 1.95±0.66 | 3.94±5.80 |
| ***IL10*, in PBMC** | 4.22±1.52 | 2.53±1.90 | 4.6±2.52 | 1.5±1.42 |
| ***TNFA*, in PBMC** | 3.94±3.03 | 3.88±3.62 | 6.31±6.06 | 1.54±1.81 |
| ***IL6*, in skin biopsy** | 2.79±1.81 | 1.49±1.61 | 1.41±1.13 | 2.28±2.14 |
| ***TNFA* in skin biopsy** | 1.14±1.51 | 1.04±0.96 | 0.46±0.22 | 1.08±0.51 |
| **miR-155-5p, in PBMC** | 1.25±0.66 | 1.34±0.38 | 2.11±0.94 | 1.16±0.67 |
| **miR-mir146a, in PBMC** | 2.86±4.04 | 1.77±0.38 | 2.01±0.86 | 1.37±0.54 |
| **miR-21-5p, in PBMC** | 2.31±1.00 | 2.53±2.34 | 1.67±1.63 | 1.77±1.41 |

CIDP, chronic inflammatory demyelinating polyneuropathy; CMT, Charcot-Marie-Tooth disease; HC, healthy control; IL, interleukin; PBMC, peripheral blood mononuclear cell; SD, standard deviation; TNF, tumor necrosis factor.
